# C-852-05. Methodological Limitations in Burn RCTs: Overestimated Effects and Inadequate Powering

**DOI:** 10.1093/jbcr/irag033.065

**Published:** 2026-04-06

**Authors:** Michelle E McCarthy, Victoria Valdes, Christian Bennett-Grobicki, Meredith Scannell, Eric Benoit

**Affiliations:** Lahey Clinic, Burlington, MA; Lahey Clinic, Burlington, MA; University of Maryland, Baltimore, MD; Lahey Clinic, Burlington, MA; Lahey Hospital & Medical Center, Burlington, MA

## Abstract

**Introduction:**

Randomized controlled trials (RCTs) are the gold standard for evaluating the efficacy of novel therapy. Trials can fail to reach significance for reasons other than ineffective interventions, leading to a type II error and rejection of potentially useful therapy. There is evidence that clinical trials in burn research are susceptible to methodological flaws such as failure to report a priori power calculations or reliance on historical control event rates that do not account for improvements in care. We hypothesized that characteristics of RCT design, such as predicted treatment effect and estimated event rate in the control group may influence the results of burn studies. We also sought to demonstrate an improvement in burn mortality over time using control group mortality as a surrogate for best medical management.

**Methods:**

Following PRISMA guidelines, we performed a systematic review of RCTs enrolling adult burn patients with mortality reported as either a primary or secondary outcome from 1978-2023. Exclusion criteria included non-RCT designs, pediatric populations, first-degree burns, and isolated ocular or gastrointestinal burns. For those trials that reported sample size assessment, we examined the difference between predicted and observed values for treatment effect and control group mortality. We also recorded information on early termination of trials.

**Results:**

Our search identified 1136 studies, of which 48 met inclusion criteria. Only 12 studies (25%) reported sample size calculation. Of the 10 trials that reported estimated treatment effect, 7 (70%) overestimated this value (Fig. 1). The mean predicted treatment effect was a 39.74% relative reduction from the control event rate, while the mean observed effect was a 2.79% reduction. Only four trials reported mortality as a primary outcome, and three (75%) of these reported an estimated mortality lower than predicted. There was a trend toward improvement in control group mortality over time in patients with 30-60% TBSA although this was non significant (Fig. 2).

**Conclusions:**

Overestimation of treatment effect and control group event rates contribute to underpowered trials that fail to reach statistical significance. As survival outcomes in burn care continue to improve, future trials must incorporate accurate contemporary data and realistic treatment effect estimations into power calculations thus minimizing type II error and decreasing the risk of prematurely abandoning potentially beneficial interventions in this complex population.

**Applicability of Research to Practice:**

As survival outcomes in burn care continue to improve, future trials must incorporate accurate contemporary data into power calculations and sample size estimates to avoid type II error and ensure that potentially beneficial interventions are not prematurely abandoned.

**Funding for the study:**

N/A.

